# Tuberculous extensor tenosynovitis of the wrist with extensor pollicis longus rupture: a case report

**DOI:** 10.1186/1752-1947-3-142

**Published:** 2009-11-20

**Authors:** Hitesh Lall, Suman Kumar Nag, Vijay Kumar Jain, Rahul Khare, Deepak Mittal

**Affiliations:** 1 Department of Orthopedics, Dr Ram Manohar Lohia Hospital, New Delhi 110001, India

## Abstract

**Introduction:**

The tendon sheaths constitute an uncommon target of extra-articular tuberculosis.

**Case presentation:**

We present a rare case of tuberculous tenosynovitis of the wrist involving the extensor tendon with rupture of the extensor pollicis longus tendon in a 55-year-old Indian man.

**Conclusion:**

Prompt surgical debridement and tissue diagnosis are essential for the diagnosis and treatment of this type of infection. With an accurate and timely diagnosis, appropriate surgery and antituberculous treatment may eradicate these infections and prevent complications.

## Introduction

Extrapulmonary tuberculous involvement of the musculoskeletal system is uncommon, accounting for only 10% of tuberculosis (TB) cases [1]. Although the tendon sheaths constitute an uncommon target of extra-articular TB, it remains the leading cause of chronic tendon sheath infection [2]. The diagnosis of tuberculous synovitis is usually delayed as it mimics many other conditions [3], which can lead to complications. Many complications of tuberculous tenosynovitis have been reported in the literature due to delayed presentation and diagnosis. We report a case of tuberculous tenosynovitis of the wrist with rupture of the extensor pollicis longus (EPL) tendon.

## Case presentation

A 55-year-old Indian man employed in the armed forces presented to us with a 5-month history of progressive painful swelling of the right wrist and inability to extend his right thumb. The swelling gradually increased in size. The pain, a mild, dull aching, had started insidiously, and gradually worsened with time. There was no associated history of fever, loss of weight or appetite, night sweats, malaise or fatigue. There was no history of trauma, pain in other joints of the body, morning stiffness of the back or hand joints, or continuous use of vibratory tools. The patient had no history of previous illness suggestive of diabetes mellitus, injuries or surgery.

Physical examination revealed a well-looking man with a right wrist swelling. The swelling was non-tender, doughy in consistency, approximately 8 × 5 cm, non-compressible, extending proximal and distal to the wrist with positive cross-fluctuation. It was mobile at right angles to the plane of the wrist joint but not longitudinally present on the dorsolateral aspect of the wrist. Movements of the wrist were only limited and slightly painful at the extremes with crepitation felt in the swelling on movement of the joint. Extension of the thumb with palm down on a table was limited and weak at the metacarpophalangeal (MCP) and interphalangeal (IP) joints indicating clinical signs of extensor pollicis longus rupture. There was no clinical evidence of chest infection or other joint involvement.

**Figure 1:**
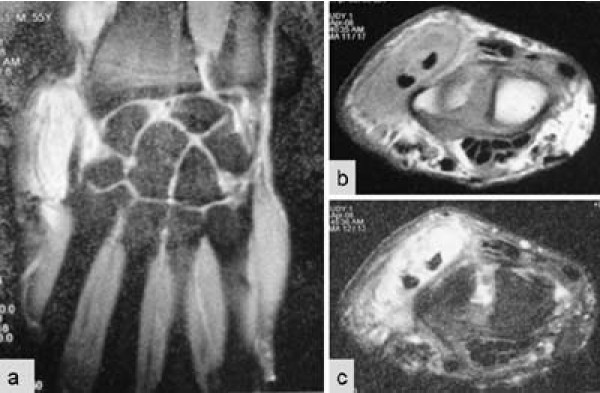
**Magnetic resonance imaging of the wrist**. (a) Coronal section proton density fat saturation; (b) axial section T1 fat saturation images showing hypointense signals on dorsolateral aspect of the wrist; (c) axial section T2 fat saturation images showing hyperintense signals on the radial side of the wrist joint surrounding the tendon.

Laboratory findings showed an erythrocyte sedimentation rate of 32 mm/hour and serum C-reactive protein was 12 mg/dl. A diagnosis of ganglion was established on fine needle aspiration cytology. Findings on chest X-rays were normal. Right wrist joint X-ray showed no significant findings. Magnetic resonance imaging (MRI) of the right wrist joint revealed tenosynovitis of the extensor tendons (Figure 1a-c).

The patient was operated on under local anesthestic and a curvilinear incision was made over the prominence of the swelling. Extensive drainage, widespread surgical debridement, irrigation, and synovectomy were performed. During the operation, multiple large melon seed bodies along the extensor tendons with adherent synovitis were found. The tendon of the extensor pollicis longus was found to be attached with a streak of fibrous tissue. The margins of the tendon were freshened and debrided and tendon transfer with extensor indices was performed. A below-elbow slab was applied with the wrist, MCP and IP joints of the thumb, in extension for 4 weeks. Intermittent mobilization with a splint during rest was applied for the following 2 weeks. Subsequently, the patient was freed of splintage and unhindered occupational activity was allowed after 3-4 months when the power in his thumb had reached near normal. Resected synovium and tissue were sent for histopathology.

**Figure 2:**
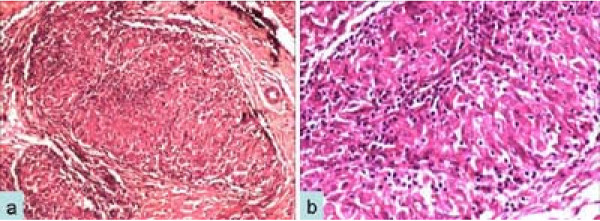
**Photomicrograph shows granulomatous inflammation, hematoxylin and eosin, 400× (a); higher magnification shows an epithelioid cell granuloma with chronic inflammation, hematoxylin and eosin, 600× (b)**.

Biopsy showed hyperplasia of the synovial lining epithelium. The underlying tissue exhibited vascular proliferation, acute and chronic inflammatory infiltrates, fibrin deposits and multiple epithelioid cell granulomas with minimal necrosis (Figure 2a, b). Ziehl-Neelsen stain was positive for acid-fast bacilli (AFB) and a positive culture for AFB was present on microbiological examination. *Mycobacterium tuberculosis *polymerase chain reaction (PCR) was positive.

Antituberculous treatment was started as soon as the identification of *M. tuberculosis *was confirmed. The patient was prescribed 300 mg of isoniazid with 10 mg of pyridoxine, 800 mg of ethambutol, 750 mg of pyrazinamide twice daily and 600 mg of rifampicin daily for 2 months. He was evaluated monthly. The lesion was completely stable and no sign of infection was detected. Consequently, treatment with isoniazid and rifampicin was planned for an additional 10 months. Postoperatively, the patient had good extension of his thumb and was able to return to the high occupational demand of the armed forces.

## Discussion

Primary tuberculous tenosynovitis is a rare condition. Tuberculous tenosynovitis selectively targets the wrist and volar aspect of the hand, where it accounts for 5% of cases of osteoarticular TB [4]. Involvement of the foot and ankle is much less commonly reported. In the wrist and hand, the flexor tendon sheath and radioulnar bursae (the compound palmar ganglion) are the most common sites of tenosynovitis. However, the digital flexor sheaths and the dorsal wrist compartment are affected less often [5-8]. Multifocal tenosynovitis has been reported recently [9]. The mechanism may be direct inoculation from adjacent bone or joint infection or seeding from a tuberculous lesion in the pleuropulmonary or genitourinary system.

The precipitating factors include trauma, overuse of the joint, old age, low socioeconomic status, malnutrition, alcoholism, immunosuppression and steroid injections. The right hand and wrist are the most common sites of involvement of tuberculous tenosynovitis and men are more affected than women tuberculous tenosynovitis of the wrist. Patients usually present with an insidious, slow-growing, sausage-like mass along the inflamed tendon with no or little pain. Patients may present with discharging sinus and carpal tunnel syndrome [6,9]. Tendon rupture is a rare presentation, but it may occur when treatment is delayed [6,10]. The onset of disease is gradual with slow progression leading to well-advanced disease before presentation, as in our patient. Mason, quoting from Kanavel's files, reports that, out of 21 available records of tuberculous tenosynovitis in the hand, rupture or impending rupture of the tendon was noted in 10 instances [11].

There are three histological forms of tuberculous tenosynovitis as a result of the long duration of the disease, the resistance of the individual, and the varying virulence of the microorganism. In the earliest stage, the tendon is replaced by vascular granulation tissue. Later on, the sheath is obliterated by fibrous tissue. Fluid is confined within the sheath and rice bodies may appear due to caseation. In the end, the tendon may consist of only a few strands of tissue and may rupture spontaneously. If healing by fibrous tissue formation fails to curtail the pathologic process, extensive caseation and granulation occur. This may lead to sinus formation and superimposed secondary infection [6,10-12]. Although granulomas (caseating and non-caseating) occur in most cases, rare cases might show nonspecific inflammation only without granulomas. "Rice bodies" or "melon seeds" represent fibrinous masses (tubercles) which are present in 50% of TB cases [12,13].

Laboratory findings are generally negative, except for the erythrocyte sedimentation rate, which is usually increased [5]. In the index case, bacteriological tests constituted the keystone of the diagnosis. MRI may show thickening of the synovial membrane with increased vascularization, fluid within the tendon sheath, reactive inflammation around the tendon, or swelling of the tendon. In contrast to acute suppurative tenosynovitis, where synovial sheath fluid is the predominant feature, relatively little synovial sheath fluid is characteristic of tuberculous pathology [13,4]. The main problem remains the difficulty in diagnosing the disease because of non-specific clinical signs that point to a number of other possibilities. Differential diagnoses of tuberculous tenosynovitis include other mycobacterial infections, pyogenic infection, brucellosis, foreign body tenosynovitis, sarcoidosis, rheumatoid arthritis, gouty arthritis, pigmented villonodular synovitis of the tendon sheath, and fungus infection [6,12-14]. Another problem is the clinical diagnosis of extensor pollicis longus rupture as the tendon may be ruptured and its continuity maintained by a flimsy fibrous tissue. The positive retroflexion sign comparing extension of the thumb toward the roof while the palms lie flat on the table, as described by Skoff, is useful to clinch the diagnosis of EPL rupture in such cases [15]. The most effective treatment involves a combination of medical and surgical therapies [6,7,11,12]. Rifampicin, isoniazid, pyrazinamide, and ethambutol are prescribed.

The currently recommended 6-month course is often inadequate and is commonly extended to 9 or 12 months. Extensive curettage, lavage and synovectomy should be performed. Surgery is essential, but the extent of surgical debridement is still debatable. Some authors advocate surgical debridement with complete excision of the tendon sheath while others advocate decompression of the tendon sheath without excision and debridement of the surrounding tissue [5,7,11,12,16]. Our patient was treated with complete debridement of involved soft tissue with reconstruction using the healthy neighbor extensor indicis tendon as the involved EPL tendon was not healthy and amenable to end-to-end repair. The patient has now had a disease-free interval of more than a year. With appropriate treatment, recovery is usually satisfactory, most notably at the extensor tendons of the hands as in our patient. TB tenosynovitis has a tendency for local recurrence, with more than 50% of cases recurring within a year of treatment [17], so close follow-up should be carried out in every case.

## Conclusion

Tuberculosis of the tendon sheaths of the wrist is rare. Delayed diagnosis is common due to slow progression and numerous differential diagnoses, which often leads to complications. Early radical excision of the infected tissues combined with antituberculous multidrug therapy gives good functional results and prevents recurrence.

## Abbreviations

TB: tuberculosis; EPL: extensor pollicis longus; MCP: metacarpophalangeal; IP: interphalangeal; MRI: magnetic resonance imaging; AFB: acid-fast bacilli; PCR: polymerase chain reaction.

## Competing interests

The authors declare that they have no competing interests.

## Authors' contributions

HL, SN, and VKJ wrote the case presentation and performed the literature review. VKJ and RK assisted in reviewing the radiology and provided images. HL and DM assisted in diagnosis. All authors read and approved the final manuscript.

## Consent

Written informed consent was obtained from the patient for publication of this case report and any accompanying images. A copy of the written consent is available for review by the Editor-in-Chief of this journal.
